# Review: pathophysiology of intracranial hypertension and noninvasive intracranial pressure monitoring

**DOI:** 10.1186/s12987-020-00201-8

**Published:** 2020-06-23

**Authors:** Nicolas Canac, Kian Jalaleddini, Samuel G. Thorpe, Corey M. Thibeault, Robert B. Hamilton

**Affiliations:** Neural Analytics Inc., Los Angeles, CA 90064 USA

## Abstract

Measurement of intracranial pressure (ICP) is crucial in the management of many neurological conditions. However, due to the invasiveness, high cost, and required expertise of available ICP monitoring techniques, many patients who could benefit from ICP monitoring do not receive it. As a result, there has been a substantial effort to explore and develop novel noninvasive ICP monitoring techniques to improve the overall clinical care of patients who may be suffering from ICP disorders. This review attempts to summarize the general pathophysiology of ICP, discuss the importance and current state of ICP monitoring, and describe the many methods that have been proposed for noninvasive ICP monitoring. These noninvasive methods can be broken down into four major categories: fluid dynamic, otic, ophthalmic, and electrophysiologic. Each category is discussed in detail along with its associated techniques and their advantages, disadvantages, and reported accuracy. A particular emphasis in this review will be dedicated to methods based on the use of transcranial Doppler ultrasound. At present, it appears that the available noninvasive methods are either not sufficiently accurate, reliable, or robust enough for widespread clinical adoption or require additional independent validation. However, several methods appear promising and through additional study and clinical validation, could eventually make their way into clinical practice.

## Introduction

Easy, accurate, and noninvasive ICP monitoring has been described as one of the holy grails of neurocritical care. Raised ICP can occur as a complication in cases of traumatic brain injury (TBI), stroke, intracranial hemorrhage, intracranial infection, hydrocephalus, brain tumor, as well as other neurological conditions [1, 2]. The direct result of elevated ICP is reduced cerebral perfusion pressure (CPP), which can result in cerebral ischemia or herniation, potentially leading to disability and increased rates of mortality [3–5].

Identifying and treating elevated ICP is imperative in the proper treatment of patients in neurocritical care settings and is essential to improving long term outcomes [6]. Raised ICP is associated with increased mortality and poor neurologic outcomes, whereas ICP monitoring of patients has been shown to improve outcome in patients with closed head injuries [7]. However, current gold standards for ICP measurement involve expensive and invasive surgeries performed by neurosurgical experts and carry a number of risks including hemorrhage, infection, and probe displacement [3]. As a result, not all patients who could benefit from ICP monitoring receive it, such as in cases where invasive monitoring is either unavailable or contraindicted, or in cases where the potential risks are determined to outweigh the benefits.

Accurate, noninvasive methods of assessing ICP would be extremely valuable, and much effort over the past several decades has been devoted to the exploration of this task. Currently, physicians can rely on qualitative features that may be suggestive of ICP pathology. These markers include absent or compressed basal cisterns, midline shift, and intracerebral hemorrhage as seen in computed tomography (CT) scans, which have been associated with increased ICP [8, 9]. These markers have only been studied in the context of head trauma and their applicability in other settings is questionable. Furthermore, even within the limited scope of head trauma injuries, the predictive value of these techniques remains unclear [10].

Though a reliable qualitative marker to distinguish normal ICP from high ICP would be useful, of even more value would be a quantitative method for measuring an individual patient’s ICP, particularly in cases where continuous monitoring would be beneficial. These methods can be broadly categorized into fluid dynamic, otic, ophthalmic, and electrophysiologic methods. The primary focus of this review will be to describe efforts to assess ICP using Transcranial Doppler (TCD) ultrasonography, a fluid dynamic method that employs measurements of cerebral blood flow velocity. A brief review of the broader landscape of invasive and noninvasive ICP measurement methods as well as a summary of the pathophysiology of ICP will also be included in this review.

## Pathophysiology of intracranial pressure

**Fig. 1:**
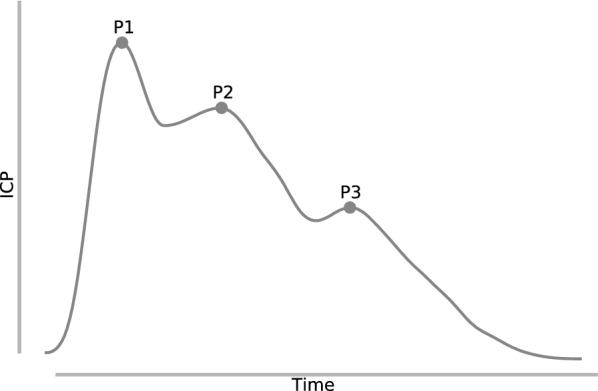
An ICP pulse waveform, with three characteristic peaks: P1, P2, and P3

In its most basic sense, ICP is the pressure inside the skull, which is reflected by the pressure of the cerebrospinal fluid. Most commonly, when we refer to ICP, we are referring to mean or static ICP. However, it bears remembering that the ICP signal is a pulsatile signal, driven by the cardiac cycle, and is typically defined by three characteristic peaks (P1, P2, and P3, as shown in Fig. 1), which can be used as a basic check when verifying acquisition of an ICP signal. Pressure changes occurring within individual pulses, often refered to as ICP pulse or wave pressure, have received relatively less study than mean ICP pressure, and so, unless otherwise specified, references to ICP should be assumed to refer to mean ICP. Normally, this mean pressure should ideally be maintained in a fairly narrow range, between about 7 and 15 mm Hg for adults, 3 and 6 mm Hg in children, and between 1.5 and 6 mm Hg in term infants [11]. Published guidelines recommend ICP $< 20-25$ mm Hg in the neurointensive care unit for TBI and other forms of acute brain injury [12, 13]. Allowing ICP to persist far outside of these ranges can have dire consequences. To understand the delicate balance involved in maintaining ICP, consider that the intracranial space contains three major components: cerebrospinal fluid (CSF), the blood supply—consisting of the network of arteries and veins that supply blood to the brain—and parenchymal tissue. These components are enclosed within an effectively rigid skull that can be treated as a closed system. Thus, pressure and volume are related: a change in volume to any one component will result in a commensurate change in ICP. This relationship between the volume of the individual intracranial components and the ICP is known as the Monro-Kellie hypothesis [14, 15]. Any major perturbation to this system risks compromising the mechanisms that continually maintain this balance.

**Fig. 2:**
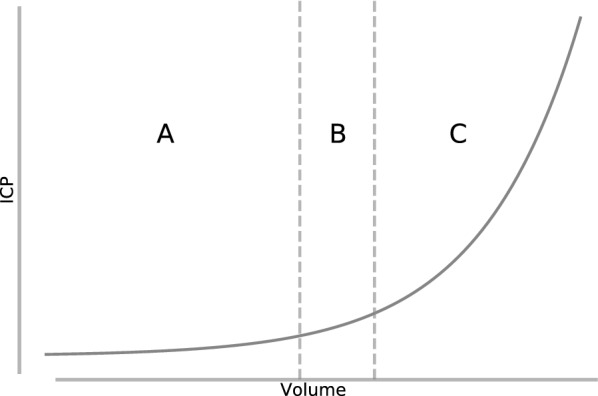
The relationship between pressure and volume within the intracranial compartment. In region A, changes in the volume of one intracranial component can be buffered by changes in the volume of other components, resulting in minimal change in ICP. In region B, this buffering capacity is becoming exhausted, and ICP, though still within a normal range, begins to rise. Finally, in region C, the buffering capacity has been completely exhausted, and ICP rises rapidly at an accelerating rate in response to an increase in one or more intracranial components

Multiple studies have explored the relationship between ICP and volume, known as the intracranial elastance curve [16–19]. They found that, within physiologically relevant ranges, the elastance curve can be well described by an exponential function:

1 $$\begin{aligned} P = P_1 e^{E_1 \cdot V}, \end{aligned}$$

where *P* is the pressure, $P_1$ is a pressure normalization coefficient, $E_1$ is a constant elastance coefficient, and *V* is the intracranial volume. This relationship is shown in Fig. 2. Initially, volume expansions by one intracranial component can be buffered by changes to the volume of the other components, particularly by the displacement of CSF or cerebral venous blood in cases of TBI and stroke (region A in Fig. 2). However, as this buffering capacity is exhausted, ICP begins to increase rapidly at an accelerating rate (region C in Fig. 2). The intracranial elastance $\Delta P / \Delta V$ serves as an indicator of the current buffering capability of the intracranial space. Though an elevated ICP generally indicates that the buffering capacity has been exhausted to a degree, a normal ICP does not necessarily mean that buffering has not already been compromised, as could be the case in region B of Fig. 2.

Because the volume of the brain is typically fixed, the two most important components contributing to ICP are the cerebral blood flow and the balance between production and absorption or outflow of the CSF. If the volume of either of these two components increases, through, e.g., intracranial hemorrhage or an inability to effectively absorb or drain CSF, without a compensatory decrease in another component, then the resulting net volume expansion will lead to increased ICP and eventually intracranial hypertension (ICH). Compensatory mechanisms exist to accomodate modest volume expansions by extrusion of the CSF or venous blood, but once these buffering mechanisms are exhausted, ICP will rise rapidly until it becomes comparable to the pressure inside cerebral arterioles. At this pressure, termed the critical closing pressure (CrCP), the arterioles will begin to collapse and blood flow to the brain ceases.

Cerebral blood flow (CBF) itself is determined by the input pressure in the form of the mean arterial blood pressure (MAP), the ICP, and the cerebrovascular resistance (CVR) according to the relationship:

2 $$\begin{aligned} CBF = \frac{MAP - ICP}{CVR} \text {.} \end{aligned}$$

A cascade of mechanisms normally operates in order to try to maintain CBF in cases of increased ICP; however, these mechanisms can in some cases result in a runaway feedback loop that results in intracranial hypertension and eventual cerebral ischemia. First, in the presence of raised ICP, distal cerebral arterioles undergo vasodilation in order to lower CVR in an attempt to offset the effects of increasing ICP on CBF. If this is not sufficient to maintain CBF, then arterial blood pressure also increases. Both of these mechanisms have the effect of increasing cerebral blood volume and, consequently, further increasing ICP, until a complete cessation of cerebral blood flow occurs. This situation is one of the primary ways in which secondary ischemic brain injury can occur in the hours or days following a primary injury and underscores the importance of ICP monitoring in neurointensive care settings [11]. Though blood flow remains relatively constant over a range of ICP on account of autoregulation, the morphology of the waveform changes. For example, the pusatility of the flow tends to increase with increasing ICP [20, 21], a feature leveraged by a number of noninvasive ICP monitoring methods, which will be discussed later.

Altered ICP can result from a number of different conditions and is dependent on a number of physiological factors including autoregulation, vessel compliance, and mean arterial pressure (MAP). The complex dependence between these different components can complicate noninvasive ICP estimation methods that rely on assumptions about the relationships between these underlying factors. For example, impaired autoregulation has been reported in head injured patients [22, 23]. Ideally, a general method of ICP measurement should not depend on the type of pathology and should be able to account for normal variability in hemodynamic variables from patient to patient.

## Invasive ICP monitoring methods

There are two primary methods of invasive ICP monitoring that are considered gold standards: external ventricular drain (EVD) or intraparenchymal probe [24]. However, both methods carry the risks of hemorrhage or infection [25, 26]. Lumbar puncture (LP) can also be used to measure ICP, and in the absence of an obstruction, LP opening pressure has been shown to correspond closely with ventricular pressure [27, 28]. However, it too is invasive, painful, and unlike other invasive methods, can only provide a snapshot of ICP, which can be problematic for disease states which exhibit varying ICP over time. Additionally, for patients that exhibit pressure differences between their spinal and intracranial spaces, the LP technique may be dangerous due to the risk of brain herniation [27, 29, 30]. As a result, LP is no longer recommended for use in diagnosing ICH in neurocritical care settings and is reserved more often for use in hydrocephalus and idiopathic intracranial hypertension [3, 29]. Guidelines regarding the accuracy of invasive ICP measurement have been outlined by The American National Standards Institute (ANSI)/Association for the Advancement of Medical Instrumentation (AAMI) and specify that ICP in the range of $0-20$ mm Hg should maintain an accuracy of $\pm 2$ mm Hg, while $ICP > 20$ mm Hg should not exceed 10% error [31, 32].

### External ventricular drainage

External ventricular drainage (EVD) is an invasive ICP monitoring system that allows drainage of cerebrospinal fluid via a catheter placed into one of the lateral ventricles via a burr hole. The EVD is connected to a pressure transducer for pressure measurements and also to a drainage system that allows for the removal of CSF as necessary. EVD is currently regarded as the gold standard for ICP monitoring owing in part to the fact that they can be continually recalibrated after placement to guard against measurement drift and due to the additional clinical value of being able to perform CSF drainage [11, 33–37]. EVD is viewed as a relatively minor surgical procedure that poses minimal risk; however, there are a number of complications that can occur, namely hemorrhagic and infectious complications. A meta-analysis that included studies which were published after 1970 and which included greater than 25 ventriculostomy procedures found that out of a total of 1,790 procedures, 5.7% of cases resulted in hemorrhagic complications with 0.61% of the total resulting in clinically significant hemorrhages [38]. Another study found that out of a total of 188 patients with EVDs, 41% showed signs of postoperative hemorrhages, but the majority of these (40 patients) were considered insignificant, punctate, or trace hemorrhages, and of the remaining larger hemorrhages, only one required surgery [39].

### Intraparenchymal probe

There are three types of microtransducer ICP monitors: fiber optic, strain gauge, and pneumatic sensors. These intraparenchymal probes have been found to be as accurate as EVDs, but unlike EVDs, they can not be recalibrated after placement, which can be a problem due to zero drift, whereas EVDs can be recalibrated at any time [12, 40]. This problem has been found in a meta-analysis to be sufficiently limited to still be considered clinically acceptable [41]. However, some work has observed significant differences in simultaneously measured mean ICP scores taken by two ICP sensors placed in close proximity so as to not be affected by intracranial pressure gradients [42, 43]. These differences are likely due to baseline pressure errors (BPE), which are marked by a spontaneous shift or drift in baseline pressure. One study observed BPEs of a magnitude that could erroneously affect patient management in 9 of 16 patients [44], though the broader risk and prevalence of BPEs is currently unknown and requires further study [32]. Placement of intraparenchymal probes also involves a surgical procedure and as a result poses similar risks to EVD; however, placement of these probes is considered less invasive than EVDs, which require ventricular puncture, so the risk of severe complications is likely lower [32].

## Noninvasive ICP monitoring methods

Noninvasive ICP monitoring methods are attractive because complications that can arise due to invasive methods, while relatively rare, are preventable. Additionally, an appropriate technique might preempt the need for some of the more cumbersome aspects inherent to current invasive measurement techniques, such as the high cost and need for neurosurgical expertise to perform the procedure. Ideally, a noninvasive ICP estimation technique would be accurate, reliable, pathology independent and capable of working on a heteregeneous patient population, use readily available equipment, and be robust to systematic differences such as operator experience. Before discussing potential noninvasive ICP monitoring methods in more detail, it bears mentioning that the goal of noninvasive ICP estimation is not necessarily to replace invasive ICP monitoring, particularly in cases where EVD is recommended due to its additional therapeutic value. Rather, noninvasive ICP estimation techniques could prove useful in scenarios such as pre-hospital triage and screening at-risk patients for potential invasive monitoring, and they should be evaluated in that context rather than as a potential replacement for invasive monitoring. The AAMI has stated that noninvasive ICP measurement differences of $\pm 2$ mm Hg for ICP between 0 and 20 mm Hg and $\pm 10\%$ for ICP $> 20$ mm Hg, relative to invasive measurement, are acceptable [32]. Though these standards appear to be quite strict given the significant advantages offered by noninvasive measurement, they reflect the potentially severe consequences of inaccurate ICP measurement on patient management. Nevertheless, the potential integration of noninvasive ICP monitoring within neurocritical care is a diverse and multifaceted space, which, as mentioned, is not necessarily intended to replace invasive monitoring. Thus, the standards for accuracy for noninvasive ICP monitoring may need to similarly reflect the diversity of potential indications, and the risk/benefit trade-offs should be weighed appropriately as the state of research continues to evolve. As the field matures, more work within the neurocritical care community will be needed in order to reach a consensus on appropriate accuracy standards for specific indications of noninvasive monitoring.

Currently, in the absence of invasive measurements, the presence of certain CT or MR features such as effacement of ventricles, sulci, and basal cisterns and significant midline shift are relied upon as signs of intracranial hypertension. However, it remains unclear how accurate or reliable these features are in the assessment of intracranial hypertension. The search for an accurate, robust, inexpensive, easy to use, and portable method for noninvasive ICP estimation is ongoing and can be broken down into several general categories that we focus on in this review: fluid dynamic, otic, ophthalmic, and electrophysiologic. Furthermore, the methods described below generally fall into one of two groups: quantitative methods that attempt to estimate the value of ICP and qualitative methods that attempt to distinguish between ICP labels (e.g., normal vs. high). Because the line dividing these two groups of methods is clearly blurred in some cases (quantitative values necessarily imply classification when the classes are numerically defined and classification often implicitly relies on quantitative estimates), the distinction is largely one of intended use as determined by the authors/researchers of each method. Nevertheless, it is important to bear this distinction in mind as both the performance metrics and standards of accuracy and precision will differ significantly depending on the suggested use case. To assist in clearly distinguishing between these two types of methods and facilitating comparisons, the following terminology will be adopted: methods that seek to determine a quantitative value for ICP will be referred to as estimation methods and methods that only attempt to identify ICP labels will be referred to as classification methods. These distinctions are listed in Table 1, which includes a broad summary of the general characteristics of each method. Since most methods can be used for either classification or estimation, the categorization here simply reflects the most common use present in the literature. Methods that are classification only are denoted as such.

A subjective ranking of each method’s current usefulness according to the authors’ assessment of current research has also been provided in Table 1. The definition of each ranking is provided in Table 2. Though higher rankings of 4 or 5 could theoretically exist for methods that have demonstrated the requisite accuracy, precision, standards of evidence, and feasibility of use to achieve widespread adoption in clinical care settings, it is the authors’ opinion that none of the current proposed noninvasive ICP monitoring methods approach this level of efficacy. This review primarily focuses on noninvasive ICP monitoring in adults, though other work has also been devoted to monitoring in children [45].

**Table 1:** General characteristics of noninvasive ICP monitoring techniques

Method	Population	Continuous	Use	Rank
TCD	Varied	Yes	Estimation	3
TDTD	Varied	No	Estimation	3
Dynamic MRI	Hydrocephalus	No	Classification	2
NIRS	TBI	Yes	Classification	1
TMD	Hydrocephalus/Meniere’s disease	No	Classification	1
OAE	Healthy	Yes	Classification	2
SVP	Unspecified	No	Classification (only)	1
ONSD	Varied	No	Classification	3
Ophthalmoscopy	TBI	No	Classification (only)	1
OCT	N/A	No	N/A	1
VEP	Varied	Possible	Estimation	2
EEG	Varied	Possible	Estimation	1

The Method column refers to the technique name or abbreviation. The Population column refers to the patient population that has been studied using that method. The Continuous column specifies whether the method can be used for continuous monitoring. The Use column refers to whether the method has been primarily indicated for use in estimation of ICP value or classification into ICP labels. Finally, the Rank column offers a subjective ranking of the authors’ assessment of the current state of research regarding the efficacy of the method for monitoring ICP: 1 (inaccurate/not useful/lack of evidence), 2 (potentially useful/needs more research), and 3 (likely useful as a supplement to invasive measurement in some situations). Abbreviations used: TCD (Transcranial Doppler), TDTD (two-depth transorbital doppler), NIRS (near-infrared spectroscopy), TMD (tympanic membrane displacement), OAE (otoacoustic emissions), SVP (spontaneous venous pulsations), ONSD (optic nerve sheath diameter), OCT (optical coherence tomography), VEP (visual evoked potentials), EEG (electroencephalography)

**Table 2:** Definitions for categorical rankings of noninvasive ICP monitoring methods

Rank	Definition
1	Method is not useful, either due to being inaccurate or simply lacking enough evidence to make an assessment
2	Method may be useful as a supplement to invasive monitoring, but results are likely very limited and mixed, and more research and/or development is needed
3	Though not necessarily universally positive, evidence is generally more consistent and substantial than for rank 2 and method is likely to be useful as a supplement to invasive measurement in at least some clinical situations in the absence of technical hurdles to adoption

### Fluid dynamic methods

#### Transcranial doppler ultrasonography

Transcranial Dopper (TCD) ultrasonography was first described by Aaslid et al. and is a tool for measuring cerebral blood flow velocity (CBFV), commonly in the middle cerebral artery [46]. Its potential use in noninvasive ICP monitoring was noted early on by Hassler et al., who observed that as ICP increased, the TCD waveform underwent characteristic changes in waveform morphology [47]. These high resistance profiles have been observed to affect flow patterns and to exhibit a progression in diastolic flow velocity that transitioned from low, to zero, to reversed, dependent on CPP [48]. Since then, TCD has received significan attention for its potential use in noninvasive ICP monitoring [49]. As a major focus of this review, TCD-based methods will be covered in detail in a later section. The majority of methods that fall under this category are estimation methods, though there are some exceptions which we will note.

#### Two-depth ophthalmic artery Doppler ultrasonography

The two-depth transorbital Doppler (TDTD) technique developed by Ragauskas et al. [50, 51] works by simultaneously measuring flow velocities in the intracranial and extracranial segments of the ophthalmic artery (OA) while applying an external pressure in a series of steps to the tissues surrounding the eyeball. The intracranial segment of the OA is subject to the pressure inside the intracranial compartment—in other words, the ICP—while the extracranial segment is subject to the externally applied pressure. The basic principle underlying this method is that when the externally applied pressure is equal to the ICP, then the measured features extracted from the flow velocity measurements in each segment should be equal to within some predefined tolerance. The majority of studies exploring the utility of this method have applied it to estimating ICP.

An initial study performed on 57 patients with severe TBI found a 95% confidence limit for prediction of invasive ICP of around 12 mm Hg [50]. A follow-up study examined a group of 62 patients of various neurologic conditions (primarily idiopathic intracranial hypertension and multiple sclerosis) with invasively measured ICP by lumbar puncture and found a bias that was not statistically different from zero, with a standard deviation of error of 2.19 mm Hg, indicating a high level of precision [51]. Several other papers have seemingly confirmed a negligible bias in a variety of conditions [52, 53]. Another paper investigated the classification efficacy of the TDTD technique by comparing it to the optic nerve sheath diameter (ONSD) method (described below) in a group of neurological patients (85 patients enrolled in TDTD and 92 in ONSD) requiring lumbar puncture and concluded that the TDTD method has better diagnostic reliability for detecting elevated ICP [54]. An independent clinical validation study looking at ICP estimation determined that the TDTD technique had fair agreement to invasively measured lumbar CSF pressure in 24 patients with normal to moderately elevated ICP, but concluded that the relatively wide Bland-Altman 95% limits of agreement of -10.5 mm Hg to +11.0 mm Hg were not sufficient to support the use of the device on its own as a measure of ICP [55]. Additionally, over 25% (15 out of 57) of the subjects that met the study criteria were excluded due to inability to insonate both segments of the OA in either eye.

The TDTD method is promising due to its relatively high reported accuracy and the fact that the method is fully automated, allowing a noninvasive ICP estimate to be taken in approximately 10 minutes [51]. However, the technique is limited in that it is unable to take continuous measurements, restricting it to cases in which only a small number of measurements are needed. The need to apply pressure to the structures of the eye for patients with certain neurologic conditions such as TBI or stroke may also be a point of hesitancy for clinicians as well as the fact that the technique requires specialized equipment [30].

#### Dynamic MRI

This method uses phase-contrast magnetic resonance (MR) imaging to measure transcranial blood and CSF volumetric flow rates. ICP can then be determined if the relationship between volume and pressure is known. A number of studies have found that, for physiologically relevant ranges of ICP, this relationship can be well approximated by the exponential function given by Eq. 1 [16]. Differentiating with respect to volume gives the so-called elastance index, a linear function of absolute ICP:

3 $$\begin{aligned} \frac{dP}{dV} = E_1 \cdot P . \end{aligned}$$

This derivative has been estimated from intracranial volume and pressure changes that occur naturally during the cardiac cycle, and the relationship has been confirmed in both human and animal studies. In addition, the elastance coefficient was found to display a relatively small degree of variability [17, 18, 56]. Currently, dynamic MRI has been primarily applied to classification of ICH, though the technique can also generate predicted ICP measurements.

Alperin et al. used five patients with time series MR images and invasively measured ICP via intraventricular catheter to derive an elastance coefficient constant, which they then used to successfully differentiate between eight healthy subjects and four patients with chronically elevated ICP [56]. They concluded that the method may provide enough sensitivity to differentiate between normal and elevated ICP, pending further study with larger sample sizes. Another study by Glick et al. examined the usefulness of this MR imaging derived ICP estimation technique in a study involving 26 symptomatic hydrocephalus patients and found that MR-derived ICP was a strong predictor of elevated ICP resolution without the need for surgical intervention [57]. Another study by Muehlmann et al. found that MR-derived ICP was positively correlated to ventriculoperitoneal shunt valve opening pressure settings in children with hydrocephalus [58].

However, Marshall et al. examined the inter- and intra-individual variability in cerebral blood flow, CSF flow, and heart rate, and the effects of these parameters on the reliability of MR-derived measurements of intracranial volume changes and elastance index [59]. In three healthy subjects, they found only modest to poor repeatability of measurements, which displayed particular sensitivity to differences in heart rate as well as requiring careful selection of representative image slices and choosing representative blood vessels. This finding is consistent with a common shortcoming observed in many methods that rely on assumptions about the relationships between hemodynamic variables, in this case ICP and elastance index. Because the relationships between the various hemodynamic inputs (e.g., heart rate, autoregulation, vessel compliance, ABP, etc.) can be highly complex, assumed simple relationships between varibles may not hold in all cases, especially over a variety of diverse pathological conditions. Further issues posed by this method are the fact that it is expensive, cumbersome, and impractical for continuous monitoring.

#### Near-infrared spectroscopy

Near-infrared spectroscopy (NIRS) is a method that can be used to estimate continuous cerebral blood volume changes by measuring changes in the local concentration of oxygenated hemoglobin in the blood [60, 61]. Weerakkody et al. studied the synchronization between ICP and NIRS variables induced by vasogenic ICP waves during CSF infusion studies in patients with TBI [62]. They found that changes in oxygenation were correlated with vasogenic ICP slow waves; however, the sensitivity of the NIRS technique to detect changes in ICP or distinguish between normal and elevated ICP states remains uncertain. Current research into this method remains preliminary, and given that studies have focused more on detecting changes rather than quantifying them, the method as currently presented appears to be aimed more at classification than estimation. In addition, the technique is limited by the availability of NIRS equipment and the fact that acquiring the required patient parameters is time-consuming and can not be done reliably in around 50% of recordings [60].

### Otic

The auditory system communicates directly with the intracranial CSF via the cochlear aqueduct, the vestibular aqueduct, and the space surrounding the auditory nerve, and thus can provide another means of noninvasive ICP estimation, as changes in ICP affect the intracochlear pressure. In this section, we describe a number of methods detailed in the literature which utilize this relationship in order to estimate ICP noninvasively.

#### Tympanic membrane displacement

The tympanic membrane displacement (TMD) technique takes advantage of the communication between the subarachnoid space and the inner ear primarily via the cochlear aqueduct which allows changes in ICP to be transmitted to the perilymph of the cochlea [63–65]. Changes in perilymphatic pressure result in movement of the inner ear ossicles causing displacement of the tympanic membrane. Measurement of this displacement serves as the basis for the TMD technique. A tympanometer can be used to detect changes in the volume of the ear canal that result from tympanic membrane displacements, and thus may serve as an indirect measure of ICP. Though early studies have suggested the TMD technique can be used to estimate ICP [65–67], most subsequent work has evaluated the method’s ability to detect changes or to differentiate between various ICP groups, so in the context of this review, we nominally view it as a classification method.

A number of studies have suggested that the TMD technique can provide useful non-invasive measurements of ICP [65, 66]. Samuel et al. reported that the TMD technique could predict changes in ICP with a sensitivity of 93% and a specificity of 100% [68]. However, a study by Shimbles et al. evaluated the TMD technique on several groups consisting of 135 hydrocephalus patients, 13 benign intracranial hypertension patients, and 77 healthy volunteers and concluded that the technique could not be used to provide reliable measurements of ICP [69]. The authors found that the technique could not even be applied in nearly two thirds of the cases within the patient population and in almost 30% of the healthy controls. Technique failure was due to failed tympanometry or lack of cochlear aqueduct patency, and the likelihood of failure was found to be affected by age, which is known to correlate with reduced cochlear aqueduct patency [70]. They also found no significant difference between the different study groups and, for the subgroup with simultaneously collected invasive ICP measurements, reported that the predictive limits of their regression analysis were an order of magnitude wider than the normal range of ICP, rendering it unsuitable as a surrogate for ICP.

A number of other studies have also found negative results associated with the TMD technique. Walsted et al. found that the TMD technique was unable to detect decreases in ICP as a result of an induced decrease in cerebral blood flow [71]. Ayache et al. determined that that TMD technique was not useful for assessing perilymphatic pressure in patients with Meniere’s disease (20 Meniere’s disease patients, 9 healthy controls) after failing to detect significant differences between the study’s groups [72].

#### Otoacoustic emission

Otoacoustic emissions (OAEs) are sounds generated by the inner ear in response to a loud sound, which can be evoked using a number of techniques [31, 73]. The mangitude of these OAEs has been shown to be sensitive to changes in ICP [74–78]. The OAEs generated within the cochlea are transmitted via the middle ear to the external ear canal, where they can be measured using low-noise microphones [79]. An advantage over other noninvasive otic ICP techniques is that the magnitude of the measured effect is generally larger due to being reduced via two passages through the middle ear, once in the forward direction and once in the reverse direction. Further, the equipment used to measure OAEs are relatively portable and easy to use. Low frequency distortion-product OAEs (DPOAE) in particular have been shown to be affected by changes in ICP resulting from changes in posture or altitude [74–76]. Only one study has compared DPOAE measurements to invasively measured ICP values. This study collected data on 18 patients grouped according to change in ICP: small ($< 4$ mm Hg), medium ($5-11$ mm Hg), and large ($\ge 15$ mm Hg) [80]. They found that significant changes in DPOAE measurements were present in only the large group. Given that the majority of studies have examined healthy subjects and looked only at detecting relative changes or differences between groups, this method has been categorized as a classification method. Notable limitations of this technique appear to be a large variability between subjects in predicted ICP values (in excess of the normal expected intersubject variability) and the fact that the method can not be applied to patients with sensorineural or conductive hearing loss [76, 81]. The method does appear to have good intrasubject reliability, however, which may make it a good candidate for periodic monitoring of relative ICP changes in patients for whom a baseline ICP has already been measured using another method.

### Ophthalmic

#### Spontaneous venous pulsations

Spontaneous venous pulsations (SVPs) are subtle variations in the retinal vein diameter seen on the optic disc. These pulsations can be assessed visually by a neuro-ophthalmologist using an ophthalmoscope or similar hand held lens. SVPs are a result of variation in the pressure gradient caused by differences between the intraocular pressure (IOP) and CSF pressure as the retinal vein traverses the lamina cribosa [82, 83]. An increase in ICP would affect this pressure gradient, and it is expected that once ICP rises above a certain threshold, SVPs should cease [84]. Thus, it has been suggested that SVPs can only be present when ICP is normal [83, 84]. Given the binary nature of assessment, this method’s primary function is classification. A study by Wong and White that examined 106 patients undergoing lumbar puncture reported a sensitivity to normal ICP of 94% based on the presence of SVP but, notably, found that patients with high ICP could indeed have SVPs [85]. Additionally, SVPs appear to be absent in about 10% of of the general population, and so their absence is not necessarily indicative of intracranial hypertension either [84]. Further, SVPs are not suitable as a method for continuous ICP monitoring due to the need for manual visual examination by an expert, and this method is further complicated by the fact that SVPs are normally evaluated in the sitting position, which can lead to a lower ICP than what would be measured in the more typical supine position [73].

#### Optic nerve sheath diameter

The subarachnoid space surrounding the optic nerve and bounded by the optic nerve sheath is filled with CSF that is contiguous with the intracranial CSF. An increase in ICP should therefore be transmitted to the CSF surrounding the optic nerve resulting in distention of the optic nerve sheath. This increase in optic nerve sheath diameter (ONSD) associated with intracranial hypertension has been reported in numerous studies [86–98]. The majority of research has examined the ONSD method as a means of classifying ICP states.

Noninvasive measurements of the ONSD can be performed using ocular ultrasound. A study by Geeraerts et al. examined 31 TBI patients requiring ICP monitoring along with 31 healthy control subjects and reported that ONSD measured with ocular ultrasound resulted in an AUC ROC of 0.96 [91]. An optimal cutoff was found around 5 mm.

Kimberly et al. examined a population of 38 patients undergoing invasive ICP monitoring and found a significant correlation between ONSD values and ICP, with an AUC ROC of 0.93 [99]. They found that the commonly used cutoff of ONSD $> 5.0$ mm yielded the best balance between sensitivity (88%) and specificity (93%) for identifying high ICP, defined as ICP $> 20\hbox { cm }\hbox {H}_{2}\hbox {O}$. A sensitivity of 100% could be achieved using a cutoff of ONSD $> 4.5$ mm, but at the cost of a specificiy of 63%. Another study by Soldatos et al. involving 50 TBI patients and 26 controls also found a significant correlation between ONSD measurements and invasive ICP values in patients with severe brain injury as determined by the Marshall and Glasgow Coma Scales. They reported an optimal cut-off value for predicting elevated ICP (ICP $> 20$ mm Hg) using ONSD was found to be 5.7 mm (74.1% sensitivity; 100% specificity).

A study by Rajajee et al. involved a heterogeneous group of 65 patients with a variety of intracranial injuries [100]. An optimal ONSD cutoff of $> 4.8$ mm was determined, resulting in a sensitivity of 96% and specificity of 94%. This study contained the largest sample, and the authors also took extra effort to obtain sharp boundaries for the optic nerve sheath and avoid contamination with previously described linear hypoechoic artifact [101]. They also emphasized controlling systematic differences and presented their case for having conducted possibly the most reliable study on ONSD and ICP to date. Additionally, they remarked on the fact that the relationship between ONSD and ICP is not expected to be linear as studies have suggested that there may be a maximum nerve sheath diameter, leading to more of an asymptotic relationship [102]. They hypothesized that differences seen in the literature could be due to the hypoechoic artifact and interobserver variability, factors which the authors placed special emphasis on controlling.

High resolution MRI has also been used to measure the optic nerve sheath [103]. Geeraerts et al. described a noninvasive method using MRI to measure ONSD [104]. A retrospective analysis of 38 patients that underwent both MRI and invasive ICP monitoring found a significant positive correlation between ONSD measured via MRI and ICP [104]. In this study, an optimal cutoff value of 5.8 mm (sensitivity 90%; specificity 92%; AUC ROC 94%) was found for detecing ICP $> 20$ mm Hg. However, this study acknowledged major limitations related to use of MRI, including limited access and specific contraindictions.

Despite some promising results, there are significant concerns with this method regarding the variability of optic nerve size due to pathology, age, etc., as well as its dependence on operator experience. Furthermore, optimal cut-off values vary widely and have been reported anywhere from 4.8 mm to 5.9 mm. While this may appear to be tightly clustered, it is important to understand the impact of this range of cutoffs. Using even a slightly different cutoff in various study populations would lead to markedly different sensitivities and specificities. For example, in Rajajee et al., they found that using the 5.9 mm cutoff reported in Geeraerts et al. would have missed 81% of the high ICP measurements using their study population. Additionally, a study investigating the relationship between ONSD and ICP measured via EVD in 20 SAH patients did not find any detectable relationship [105]. Further, in 10 of the patients, changes in ONSD were monitored during fairly rapid ICP change after controlled CSF drainage. Only two patients displayed agreement between the profiles of ICP and ONSD in both eyes, while four showed agreement in one eye, and the remainder showed no agreement, leading the authors to conclude that ONSD measurements can not be used to accurately estimate ICP in SAH patients [105].

Though this method is relatively easy, has readily available equipment, low cost, and high temporal resolution, it is not suitable for continuous monitoring and instead needs to be repeated at regular intervals for at-risk patients. At best, it is likely that the ONSD method will only be a complement to invasive monitoring and will not be able to replace it. Additionally, MRI may provide more precise measurements compared to ultrasound, but it carries its own set of disadvantages [106]. However, at least one study has found good agreement between ultrasound and MRI measured ONSD [105]. Overall, the ONSD method may be useful for classification (high vs. low), but has not been demonstrated to be useful for assessing the degree of intracranial hypertension or measuring ICP.

#### Ophthalmoscopy

Papilledema may result from elevated ICP in cases of acute head injury and can be identified by ophthalmoscopy and evaluated qualitatively according to the Frisen Scale into 5 categories [107]. As papilledema can be seen as a sign of increased ICP, it is thought that ophthalmoscopy may be able to be used as an early screening/classification method in cases of suspected raised ICP. However, the grading scale is not widely applicable or accepted and its application depends heavily on the expertise of the examiner as well as requiring good visualization of the optic disc [108]. Additionally, optic disc swelling can occur slowly, making this method unsuitable in cases where a sudden increase in ICP may occur [107]. Furthermore, precisely how papilledema evaluations correlate to changes in ICP remains unknown, and studies exploring the relationship between papilledema and invasively measured ICP are currently lacking.

#### Optical coherence tomography

Optical coherence tomography (OCT) is an imaging technique that acts effectively as an “optical ultrasound” and can be used to measure retinal nerve fiber layer (RNFL) thickness in papilledema [109]. Intracranial hypertension can result in swelling of the RNFL [31]. A patented method exists that uses OCT to measure RNFL thickness and thereby infer ICP values [110]. However, its practical usefulness in clinical practice is limited by a number of factors: OCT algorithms can fail when optic disc edema is severe, determining the cause of reduced RNFL thickness—whether due to edema improvement or simply optic nerve atrophy—may not be possible, and the fact that the rate of disc edema is typically very slow [73]. Further, there is limited evidence to support any claims regarding the exact relationship between RNFL thickness and ICP [31]. Kupersmith et al. suggested that using OCT to identify deflection of the peripapillary retinal pigment epithelium (RPE) and Bruch’s membrane angle could also be used to evaluate papilledema [111]. However, once again, determining how these qualitative evaluations correlate to ICP still needs to be studied. Given the limited state of research into the relationship between OCT and ICP, it can not currently be considered a plausible ICP monitoring method, for either classification or estimation.

### Electrophysiologic

#### Visual evoked potentials

Visual evoked potentials (VEP) are measurements of the electrical response to some kind of visual stimulus as measured by placing electrodes on the back of the head over the occipital cortex. Two early studies suggested a relatively strong linear relationship ($R^2 \approx 0.7$) between VEP N$_2$ wave latency and ICP [112, 113]. Follow-up studies have further examined the relationship between VEP and ICP and its ability to estimate ICP. Zhao et al. also reported a strong correlation between flash visual evoked potential (FVEP) latency period and invasive ICP measured via either lumbar puncture or cerebral epidural manometric methods in a study involving 152 patients with intracranial pathology given mannitol injection [114]. They reported a mean relative error of $13.2\%$ and a $95\%$ confidence limit of 8 mm Hg. A limitation of these studies was the exclusion of patients exhibiting any of the following conditions: hypophyseal tumor, hypoxia, liver dysfunction, uremia, severe acidosis, and diseases affecting visual acuity. Other studies have also suggested a correlation between VEP alterations and elevated ICP [115, 116]. One study investigated the use of a device at two hospitals that combines FVEP and TCD based ICP estimation methods and showed that this instrument was also correlated with ICP while overcoming some of the shortcomings of either method individually [117].

Additional limitations of the VEP method include its unsuitability for patients with bifrontal hematoma, retinal concussion, or contusion of the optic nerve due to inaccurate measurement of FVEP value in these cases [114]. Additionally, the VEP method is difficult to use for continuous monitoring and requires a high degree of neurophysiological expertise. Andersson et al. suggested that there exists a high degree of variability in terms of latency, amplitude, and waveform across subjects, and that this variability renders FVEP an unreliable method for noninvasive ICP estimation [118].

#### Electroencephalography

Chen et al. used electroencephalography (EEG) power spectrum analysis to noninvasively estimate ICP [119]. They recorded EEG signals in 62 patients with varied CNS disorders and performed EEG power spectrum analysis. They found a significant negative correlation ($r\,=\,-\,0.849$; $p<0.01$) between EEG derived intracranial pressure index and ICP measured via lumbar puncture but did not report bias and precision. Though possible, EEG is difficult to use for continuous monitoring for long periods of time, is cumbersome to use in an emergency care setting, and its reliability and accuracy as a noninvasive ICP estimator remains to be demonstrated.

### TCD-based methods

A comprehensive literature search was performed using the search engine PubMed with the goal of identifying TCD-based methods for noninvasive ICP assessment that have been tested against invasively measured ICP in human adults. The following search criteria was used:

(intracranial pressure[Title/Abstract] OR ICP[Title/Abstract] ORintracranial hypertension[Title/Abstract] OR cerebral perfusionpressure[Title/Abstract]) AND (noninvasive OR non-invasive) AND(flow velocity[Title/Abstract] OR TCD[Title/Abstract] OR transcranial doppler).

This search phrase consisted of three blocks. The first block was aimed at capturing papers that measured ICP or quantities closely related to ICP, such as intracranial hypertension or cerebral perfusion pressure. The second block captures the requirement for noninvasive ICP estimation methods, and the final block captures the requirement that the method be based on TCD or cerebral blood flow measurements. Most terms were searched for within the Title/Abstract field in order to maintain a manageable number of total search results. Additionally, only articles written in English were considered. At the time of writing in November 2019, this search produced 249 results, which were then manually curated in order to identify qualifying publications.

The scope of this section of the review was restricted to publications that describe noninvasive methods for estimating ICP that utilize TCD measurements from the middle cerebral artery (MCA). Qualifying publications must also have included comparisons of noninvasively measured ICP against simultaneously recorded invasive ICP measurements in human adults. For this section, other review papers were also excluded as their content should be derivative of the individual publications already included. The list of methods resulting from this literature search and a summary of their results are presented below. Because of the large number of noninvasive ICP monitoring techniques that have been investigated using TCD, these methods will be further broken down into three main categories: methods based on pulsatility index (PI), methods based on estimation of CPP, and model-based methods.

For each category, a summary of the studies for each method are included as a table. For each study, a categorical “usability score” has been assigned according to the authors’ assessment of the research. This score is meant to broadly indicate how well the method performed in each particular study in terms of classification and/or estimation of ICP. The general interpretations for these scores are provided in Table 3. For example, a classification usability score of 1 would indicate that in that specific study, the accuracy obtained for the method would effectively be of no use for the classification of ICP in a clinical setting. We stress that these scores reflect only the results of each individual study and should not by themselves be broadly applied to the method that they test: one should not interpret a strong score for one study as saying that the method itself is strong. These scores have been tabulated in this way simply to provide a way of evaluating, at a glance, the amount, quality, and consistency of results for a particular method, and the broader evaluation of a method should be based on the level of accuracy and consistency across a relatively large number of studies. As one final note, the quantitative examples provided in Table 3 are not meant as rigid numerical cutoffs, but instead as rough guidelines to assist in interpreting the scores. Furthermore, the example metrics clearly do not include all possible performance metrics; rather, they are meant to represent some of the more common and easily interpretable measures used in assessing ICP monitoring methods. The usability scores provided are intended to take into account all relevant aspects of the research as a whole, which include but are not necessarily limited to, such metrics.

**Table 3:** Descriptions of usability scores for classification and estimation of ICP, as well as approximate quantitative examples corresponding to each score

Score	Description	Examples
1	None	$|r| \le 0.25$; AUC around 0.5; bias and SD $>10$ mm Hg
2	Weak	$0.25 < |r| \le 0.5$; AUC $0.55-0.7$; bias and SD $<10$ mm Hg
3	Moderate	$0.5 < |r| \le 0.8$; AUC $0.7-0.9$; bias and SD $<4$ mm Hg
4	Strong	$|r| > 0.8$; AUC above 0.9; bias and SD $<2$ mm Hg

#### PI-based methods

**Fig. 3:**
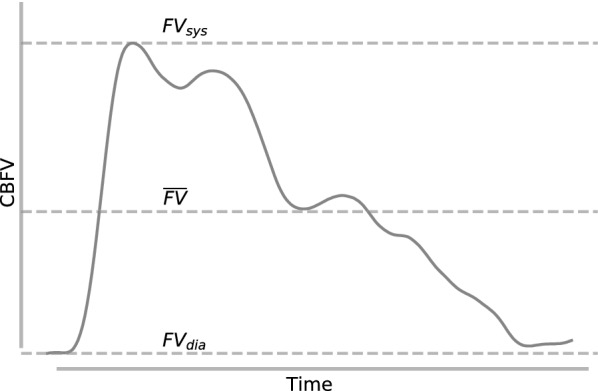
CBFV pulse waveform. Systolic, diastolic, and mean flow velocity are used to calculate PI

Like the ICP waveform, the CBFV waveform is also a pulsatile signal driven by the cardiac cycle. A single pulse is shown in Fig. 3. The Gosling pulsatility index (PI) describes the pulsatility of a CBFV waveform and is often interpreted as a measure of distal cerebrovascular resistance (CVR) [120–122]. As PI is normalized to mean velocity, it has the advantage of being insensitive to changes in measured velocity, which can vary dramatically based on vessel size and insonation angle.Mathematically, it is defined as the difference between the systolic and diastolic flow velocities divided by the mean flow velocity:

4 $$\begin{aligned} \text {PI} = \frac{\text {FV}_{sys} - \text {FV}_{dia}}{\overline{\text {FV}}} . \end{aligned}$$

Despite its common interpretation simply as a reflection of CVR, more recent hypotheses have pushed for a view of PI as a much more complex function of various hemodynamic factors [123]. For example, de Riva et al. explored two clinical scenarios—intracranial hypertension and hypocapnia—where opposite changes in CVR both led to an increase in PI. In particular, during ICP plateau waves (in which ICP increases suddenly above 50 mm Hg and lasts longer than 5 minutes before returning to normal), vasodilation led to a decrease in CVR whereas during hypocapnia, vascular constriction led to an increase in CVR. In both cases, however, PI was found to increase. In response to this observation, they concluded that PI is ultimately the product of the relationship between CPP, arterial pressure pulse amplitude, CVR, arterial compliance, and heart rate, and that it is a better indicator of CPP as opposed to ICP.

Nevertheless, models also provide some theoretical basis for the ICP-PI relationship in certain conditions. Under normal conditions, PI is predicted to increase linearly with ICP [20, 21]. However, alterations to cerebral autoregulatory strength, vessel compliance, mean arterial pressure, and the state of intracranial dynamics specific to various neuropathological conditions can in some cases radically affect the slope, offset, and even linearity of the relationship. Thus, it should not be expected that the same relationship ought to hold for all patients across a wide range of conditions, casting doubt on the practical reliability of using PI as an indicator of ICP.

Fundamentally, methods that seek to use PI to estimate ICP attempt to do so by using linear regression to model the relationship between the two variables:

5 $$\begin{aligned} ICP = a \cdot PI + b , \end{aligned}$$

where *a* and *b* are coefficients that must be estimated from the sample data. A large number of publications have attempted to estimate the value of the regression coefficients and apply their findings to the problem of noninvasive ICP estimation [124–138]. A summary of such publications is provided in Table 4.

**Table 4:** Studies exploring PI-based TCD method

References	Population	Classification use score	Estimation use score
Gao [124]	TBI, 43; hemorrhagic stroke, 7	2	1
Prunet [126]	TBI/stroke/SAH, 20 Control, 20	4	–
Robba [127]	TBI/polytrauma/SAH, 22	1	–
Robba [128]	TBI/SAH/intracranial hemorrhage, 64	1	–
Voulgaris [129]	TBI, 37	3	–
Wakerley [130]	Varied, 78	3	–
Wang [131]	TBI, 75; hypertensive brain injury, 15; intracranial lesions, 3	4	–
Zweifel [132]	TBI, 290	2	1
Bellner [133]	TBI/SAH/other, 81	4	3
Moreno [134]	TBI, 125	4	–
Brandi [135]	TBI, 45	–	2
Behrens [136]	INPH, 10	–	1
Rainov [137]	Hydrocephalus, 29	2	1
Rajajee [138]	ALF, 21	1	–
Park [125]	TBI, 11	2	3

Refer to Table 3 for interpretation of scores. If a method was not evaluated in the context of either classification or evaluation, then no score is provided for that use case

Though PI has been found to correlate with invasively measured ICP with reported accuracies as high as $\pm 4.2 \text { mm Hg}$ for ICP values between 5 and $40 \text { mm Hg}$ [133], a wider examination of available studies suggests that, across a broad range of possible scenarios and conditions, PI alone may be plagued by poor precision and a level of inconsistency and variation that have made it unsuitable thus far for clinical adoption. For example, Behrens et al. used lumbar infusion to artificially alter ICP within a group of 10 individuals with idiopathic normal pressure hydrocephalus and found that the $95\%$ prediction interval given a particular PI was unacceptably large—on the order of $\pm 25 \text { mm Hg}$—and thus concluded that PI alone is not an accurate method to assess ICP [136]. Additionally, Shen et al. examined the inter- and intra-technician variability of measured peak systolic and end diastolic cerebral blood flow velocities within the MCA and determined that while a high level of reproducibility is possible, lack of regular practice can significantly reduce the accuracy and reproducibility of measurements [139]. Based on the current state of research, it appears that PI by itself is likely too limited to be of broad clinical use as a means of estimating ICP across a range of neurological conditions. However, extreme values of PI may be useful for supporting the decision to begin invasive ICP monitoring.

A natural extension of PI-based methods is to investigate whether the linear model can be improved by including additional hemodynamic variables in the linear regression. The authors in [140] investigated this idea by constructing a multivariable linear model that included hematocrit, mean ABP, heart rate, and arterial $\hbox {CO}_{2}$ pressure, but they concluded that PI was not a sufficiently strong predictor of ICP on its own and that its predictive reliability did not improve significantly with the inclusion of additional hemodynamic variables. They attributed this to the fact that there are too many dynamic variables in the injured brain that can not be properly accounted for in such a constrained model.

#### CPP-based methods

CPP based noninvasive ICP methods rely on the assumption that ICP can be calculated as the difference between arterial blood pressure (ABP) and cerebral perfusion pressure:

6 $$\begin{aligned} ICP = ABP - CPP . \end{aligned}$$

In these methods, CPP, rather than ICP, is estimated, and, combined with independent ABP measurements, which can be performed noninvasively or minimally invasively [141], ICP can then be calculated. A number of formulas have been proposed which can be used to estimate CPP (CPPe) based on noninvasively measured signals, which are listed below [142–145]. A summary of research exploring these formulas is provided in Table 5.

**Table 5:** Studies exploring CPP-based TCD methods

Method	References	Population	Classification use score	Estimation use score
Aaslid formula (Eq. 7) [142]	Aaslid 1986 [142]	Hydrocephalus, 10	3	2
	Czosnyka [143]	TBI, 96	–	2
Czosnyka formula (Eq. 8) [143]	Czosnyka [143]	TBI, 96	–	2
	Schmidt [146]	TBI, 25	–	2
	Brandi [135]	TBI, 45	–	2
	Cardim [147]	TBI, 40	2	2
	Cardim [148]	Hydrocephalus, 53	–	1
Edouard formula (Eq. 9) [144]	Edouard [144]	TBI, 20	–	1
	Brandi [135]	TBI, 45	–	1
Varsos formula [145] (Eq. 10) [145]	Varsos [145]	TBI, 280	4	2
	Cardim [147]	TBI, 40	3	2
	Cardim [148]	Hydrocephalus, 53	–	1

Refer to Table 3 for interpretation of scores. If a method was not evaluated in the context of either classification or evaluation, then no score is provided for that use case

Aaslid et al. proposed the following formula:

7 $$\begin{aligned} CPPe = (V_0/V_1) \cdot ABP_1 , \end{aligned}$$

where $V_0$ is the mean flow velocity, $V_1$ is the amplitude of the first harmonic of the velocity waveform, and $ABP_1$ is the amplitude of the first harmonic of arterial pressure [142]. This formula was based on the expectation that the ratio of mean flow to the pulsatile amplitude of flow should be roughly proportionally related to CPP after trying to approximately account for changes in the pulsatile amplitude of the arterial pressure waveform. The relationship assumes that the effect of compliance and ICP pulsatility on CPP are negligible, and the authors adopt the approach that this formula and its underlying assumptions are a hypothesis to be tested empirically.

Another formula that was proposed based on specific observed patterns seen in TCD waveforms is

8 $$\begin{aligned} CPPe = ABP_m \cdot \frac{FV_d}{FV_m} + 14 \text { mm Hg,} \end{aligned}$$

where the subscripts *m* and *d* denote mean and diastole, respectively, and where the 14 mm Hg constant term represents a calibration parameter determined using a maximum likelihood method [143, 146, 149, 150]. Similarly to the Aaslid et al. formula, this formula is also generally unable to compensate for changes in vascular resistance, and therefore relies on these effects to be small, an assumption that may not always hold, as in periods of hyperventilation, for example.

Edouard et al. suggested a formula combining the phasic values of flow velocities and arterial pressure:

9 $$\begin{aligned} CPPe = \frac{FV_m}{FV_m - FV_d} \cdot (ABP_m - ABP_d) \text {,} \end{aligned}$$

where once again *m* and *d* indicate mean and diastole, respectively [144]. This formula was originally suggested for use as a method of assessing CPP in pregnant women [151]. It is based on the Aaslid et al. formula, but substitutes approximations for the area under the pulsatile amplitude of the flow velocity and ABP waveforms for the first harmonic of the velocity and pressure recordings.

A final formula is based on the critical closing pressure (CrCP), which represents a threshold of ABP below which blood pressure in the cerebral microvasculature is insufficient to prevent the collapse of the vessel and subsequent cessation of flow [152]. The equation for estimated CPP is:

10 $$\begin{aligned} CPPe = ABP \left( 0.734 - \frac{0.266}{\sqrt{(CVR \cdot Ca \cdot HR \cdot 2 \pi )^2 + 1}} \right) - 7.026 , \end{aligned}$$

where CVR and Ca denote noninvasive cerebrovascular resistance and arterial compliance, respectively, and HR is the heart rate [145, 153]. This formula was derived from an electrical circuit model of the cerebrovascular bed, which treated cerebrovascular resistance and cerebrovascular compliance as parallel resistive and capacitive elements, respectively [153]. The constant coefficients were derived by fitting the formula according to an analysis of a database of 232 retrospective TBI cases [145].

The reported accuracy of these methods for estimating CPP varied between $\pm 12$ and $\pm 48.9$ mm Hg, and from $\pm 12$ to $\pm 59.6$ mm Hg for noninvasive ICP estimation, with the method based on CrCP yielding the best accuracy. More study may be needed, but currently, these methods do not appear to achieve the level of accuracy necessary for achieving widespread clinical adoption. All of the formulas presented here rely on simplifying assumptions about the magnitude of the effect of various hemodynamic components, and thus should not necessarily be expected to hold in all cases where extreme values for the inputs or outputs are expected, where cerebral abnormalities or pathological conditions are present, or where the impact of confounding variables such as heart rate are unknown. This reliance on underlying assumptions represents a central challenge for methods based on simple formulas such as the ones presented here, and their use should be restricted to the specific conditions under which they have been empirically validated.

#### Model-based methods

In the context of this review, model-based methods are effectively any method that uses a model more complicated than the simple linear models and formulas described in the previous sections. This category of methods is also the broadest category and can generally be broken down into two types of methods: theory-based and data-based methods. Theory-based methods typically involve some mathematical model designed to simulate intracranial states based on some initial state, boundary conditions, model parameters, and observable measurements. These hemo- and hydro-dynamic models of the intracranial fluid dynamics are based on physical principles and have the advantage of not being wholly dependent on collecting a large amount of training data; however, they can be highly complex and their usefulness is not obvious in the absence of significant amounts of empirical validation.

Data-based methods are more common and rely on a large amount of training data that faithfully represents the target patient population in order to properly fit the model parameters. These methods tend to be more “black box” in nature, which is an advantage in that they do not require a detailed and accurate theoretical understanding of the complex underlying physics that govern the intracranial dynamics. In principle, the relevant relationships can be learned by the model; however, the drawback of these types of approaches is that the validity of the resulting model is heavily dependent on having a large amount of appropriate training data due to the complex nature of the underlying physiology and the variation between individual subjects. This is a significant issue as ICP data is inherently limited due to the invasive nature of ICP measurement and the difficulty in obtaining consistent, high-quality data. In an effort to retain the best of both worlds, some methods have attempted to combine aspects of theory-based and data-based models. A summary of the research into model-based methods is provided in Table 6.

**Table 6:** Studies exploring model-based TCD methods

Method	References	Population	Classification use score	Estimation use score
Data-based models				
Schmidt system ID model [154]	Schmidt [154]	TBI, 11	–	2
	Schmidt [155]	TBI, 17	–	2
	Schmidt [156]	Hydrocephalus, 21	–	3
	Budohoski [157]	TBI, 292	–	2
	Cardim [147]	TBI, 40	2	2
	Cardim [148]	Hydrocephalus, 53	–	1
	Schmidt [158]	TBI, 137	–	3
	Schmidt [159]	Varied cerebral diseases, 41	–	2
SCA Schmidt model [160]	Schmidt [160]	TBI, 135; hemorrhagic stroke, 10	–	2
Schmidt fuzzy pattern model [161]	Schmidt [161]	TBI, 103	3	2
Calibrated Schmidt model [162]	Schmidt [162]	Brain lesions, 13	–	2
	Schmidt [159]	Varied cerebral diseases, 41	–	2
Nonlinear Schmidt model [163]	Xu [163]	TBI, 14; hydrocephalus, 9	–	2
Data mining [164]	Hu [164]	TBI, 9	–	2
	Kim [165]	TBI, 57	–	2
Ensemble sparse classifiers [166]	Kim [166]	TBI, 80	2	–
Semisupervised learning model [167]	Kim [167]	TBI/SAH/NPH, 90	4	–
Linear discriminant analysis [168]	Aggarwal [168]	ALF, 16	2	–
SVM [169]	Chacon [169]	TBI, 8	–	4
Random forest [170]	Wadehn [170]	TBI, 36	3	–
Theory-based models				
Kashif model [171]	Kashif [171]	TBI, 37	3	3
	Park [125]	TBI, 11	2	3
Pressure corrected Kashif model [172]	Noraky [172]	SAH, 5	–	3
DC Kashif model [125]	Park [125]	TBI, 11	3	3
Hybrid models				
Hybrid model	Wang [173]	SAH/TBI, 2	–	4

Refer to Table 3 for interpretation of scores. If a method was not evaluated in the context of either classification or evaluation, then no score is provided for that use case

Due to the somewhat complicated interplay between ICP, arterial pressure, and cerebral hemodynamics, a large number of methods have attempted to incorporate arterial pressure measurements, which may provide complementary information to assist in measuring ICP [125, 154, 155, 157–163, 169–175]. These methods are not technically noninvasive as they require the placement of a radial artery catheter for monitoring ABP; however, this procedure is typically already performed as part of standard care in neurointesive care units, and the risks associated with monitoring ABP via radial artery catheter are considered significantly less risky than invasive ICP monitoring. The potential utility of this method was explored early on in a data-based method by Schmidt et al. [154], in which a system identification procedure was used to estimate ICP from ABP and CBFV measurements. They reported a bias of $4.0 \pm 1.8 \text { mm Hg}$ between predicted and measured ICP in a sample of 11 intensive care patients with epidural ICP monitoring device. A follow up study involving a group of 17 severely head-injured patients with invasively measured ICP also found that the method had the ability to predict dynamic changes in ICP, with a reported bias of $8.3 \pm 5.4$ mm Hg at baseline and $7.9 \pm 4.3$ mm Hg at the top of plateau waves [155]. However, follow-up efforts with much larger study groups consisting of TBI patients concluded that, while this method could estimate ICP with moderate accuracy, the relatively wide prediction interval (as high as 17 mm Hg) meant that this method alone was still insufficient for broad clinical application [157, 158].

Significant additional work has been done to explore ways of improving this black box method. It was found that including patient specific calibration, performed in a number of different ways, could be used to improve the accuracy of ICP estimation [159, 162]. This result that including patient specific data in a data-based model improves accuracy seems somewhat unsurprising, but also of limited utility, as one of the common goals of estimating ICP noninvasively is to do so without the need for patient specific calibration. Another method attempted to dynamically incorporate estimates of the state of cerebral autoregulation (SCA) into the model [160]. Using this procedure, the authors found that the bias of the ICP estimation model decreased significantly compared with not including SCA information, from 7.6 mm Hg to 6.9 mm Hg. To deal with the complexity of estimating ICP for a hetereogeneous patient population, Schmidt et al. used a fuzzy pattern classification method to map identified clusters within the patient parameter space to a variety of local estimators [161]. However, they concluded that none of the models showed a statistically significant improvement over the linear black box model. Another approach relaxed the assumption of a linear relationship between ABP, ICP, and CBFV and instead adopted nonlinear kernel regression approaches, which resulted in a statistically significant reduction in bias for the test dataset from 6.7 to 6.0 mm Hg [163].

Hu et al. proposed a general data mining framework for noninvasive ICP assessment which used a database composed of simultaneously recorded ABP, CBFV, and ICP measurements to achieve improved median normalized prediction error and median correlation coefficient (39% and 0.80, respectively) between estimated and measured normalized ICP when compared to the system identification method used by Schmidt et al. (51% and 0.35) [164]. Further work explored different linear and nonlinear mapping functions to identify how the performance of their data mining approach could be improved and found that nonlinear mapping functions could improve noninvasive ICP estimation over linear functions [165]. Another study used a classification technique known as Ensemble Sparse Classifiers to diagnose intracranial hypertension in head-injured patients using morphological features extracted from CBFV waveforms [166]. Kim et al. proposed a method also based on the morphological analysis of CBFV waveforms using both supervised and semi-supervised learning approaches and reported predictive accuracies as high as 82% and 92%, respectively, in classifying normal vs. hypertensive intracranial pressure [167]. Additional data-based learning techniques including the use of support vector machines, linear discriminant analysis, and random forests using features extracted from ABP and CBFV waveforms have also been shown to achieve low error and promising classification accuracies in isolated cases [168–170].

All of the model-based TCD methods discussed thus far have been considered data-based models, which do not require a detailed understanding of the physiology as an input assumption. These models implicitly rely on the assumption that information or features extracted from the TCD waveform are causally related to ICP and that the potentially complicated, nonlinear relationships that may depend on a whole host of physiological variables can then be learned by the model. While the amount of promising results suggests that this assumption is likely true to good approximation, due to the lack of underlying physiological basis, significant additional independent validation is required to demonstrate clinical utility and determine the conditions that need to be satisfied for various models to be valid.

In contrast to data-based models, theory-based models do attempt to model the physiological relationships based on a priori knowledge. One such approach to ICP estimation attempts to model the physiological relationships between ABP, CBF, and ICP using an electrical circuit analog, where pressures are represented by voltages and flows by currents, which we refer to as the Kashif model [171]. The resistance and compliance of the cerebral vasculature are represented by single resistance (R) and capacitance (C) elements, respectively. The model simultaneously estimates ICP along with R and C by requiring the model constraints to be satisfied as closely as possible according to the obtained measurements over an estimation window consisting of at least five consecutive beats and under the assumption that ICP, R, and C are constant over that window. In practice, ABP measured at the radial artery is used in place of cerebral ABP and CBFV is used as a surrogate for CBF. Care is required to properly scale and time align the signals in order to accurately approximate the actual phase relationship between cerebral ABP and CBF. This theory-based modeling approach was validated on a sample of 37 patients with traumatic brain injury and concurrently measured invasive ICP, achieving a bias of 1.6 mm Hg and a standard deviation of error (SDE) of 7.6 mm Hg. Averaging bilateral ICP estimates reduced the bias to 1.5 mm Hg and SDE to 5.9 mm Hg.

A number of models have attempted to build upon the Kashif model. One refinement attempted to correct for hydrostatic fluid pressure differences associated with the different locations between the ABP and ICP pressure transducers by adjusting the ABP to account for the vertical height between the two pressure measurement locations [172]. On a sample population of five SAH patients from which 28 data recordings were extracted, the researchers obtained a bias of $-0.7$ mm Hg and a SDE of 4.0 mm Hg. A different, simplified circuit model was developed by Lee et al. by considering only the “direct current” (DC) components of the inputs [174]. In this way, the capacitance element can be ignored as compliances have infinite impedance when the input is DC only, resulting in a model that consists of two simple resistance (SR) circuits, each made up of a single voltage source and single resistor, for estimating ICP. This SR method does not require a long window and thus is more appropriate for detecting abrupt changes in ICP. However, though this method appeared to successfully track sudden ICP changes, it was only tested on simulated data and on patients performing Valsalva maneuver. Additionally, it did not include an adaptive algorithm for estimating model parameters such as the resistance of intracranial arteries, which account for the effects of autoregulation. To solve these issues, a follow-up study was conducted which employed an unscented Kalman filter to perform adaptive internal state estimation and validated the method on 11 TBI patients, obtaining a bias of 0.21 mm Hg and SDE of 3.52 mm Hg, which compared favorably relative to the Kashif model and the PI-based method [125]. These theory-based models may appear more attractive in that they are physiologically motivated rather than just the result of fitting some algorithmic model; however, the consequence of this approach is that they must contend with deciphering the complex dynamics of the cerebral vasculature. In order to do this, theory-based models must make simplifying assumptions that ignore some of the effects of variables such as vessel compliance, heart rate, and autoregulatory strength, which may become meaningful under exactly the kinds of extreme conditions these methods are expected to diagnose.

Attempting to combine aspects of both theory-based and data-based methods, Wang et al. adopted a previously developed multiscale cerebrovascular model to simulate hidden intracranial states [173, 176]. They then integrated patient specific TCD-based CBFV data into the model using a Bayesian data assimilation framework that employed a regularizing iterative ensemble Kalman filtering method to tune model parameters and calibrate ICP prediction [173]. This method was again validated initially against synthetic data before feasibility was demonstrated on two patients undergoing invasive ICP monitoring via EVD. In both patients, prediction accuracy increased after assimilating CBFV data into the model and the researchers obtained a prediction error for mean ICP of less than 2 mm Hg in each patient, within the clinically accepted standard [31, 32, 177]. However, significantly more work involving larger, heteregenous patient populations needs to be conducted in order to establish the efficacy of this method for ICP estimation.

#### Disadvantages of TCD methods

Though numerous studies have suggested promising results for the use of TCD in noninvasive ICP monitoring, additional study is needed to show that it has the requisite accuracy for clinical use, and there remain notable obstacles to its more widespread adoption. TCD has historically required a skilled technician and has shown both intra and inter-operator variability [139]. Additionally, it has been estimated that around 5–15% of patients may not have a valid transtemporal window; however, more studies are needed [47, 178, 179].

## Conclusion

ICP monitoring is a critically important component of proper neurocritical care and management of patients with acute brain injuries in order to prevent secondary insult and the potentially severe complications that can result. Unfortunately, the procedures for monitoring ICP are invasive and carry their own sets of risks, and as a result, not all patients that could benefit from ICP monitoring receive it. As such, significant efforts have been made to develop a method for monitoring ICP noninvasively. Such a method would not necessarily replace invasive monitoring but could be used for pre-hospital triage, monitoring at-risk patients to assess the need for invasive monitoring, and in cases where invasive monitoring is deemed too risky or is otherwise contraindicted by other factors. To date, no method appears to have achieved the level of accuracy, reliability, and independent validation necessary for widespread clinical acceptance. However, a number of methods appear to hold promise and remain the subject of active, ongoing research. Of particular focus in this review were TCD-based methods, which are especially attractive due to the low cost, portability, and high temporal resolution of TCD.
